# Assessing the Association between Oral Hygiene and Preterm Birth by Quantitative Light-Induced Fluorescence

**DOI:** 10.1155/2014/374694

**Published:** 2014-01-05

**Authors:** Christopher K. Hope, Qian Wang, Girvan Burnside, Adejumoke A. Adeyemi, Siobhan Quenby, Philip W. Smith, Susan M. Higham, Melissa Whitworth

**Affiliations:** ^1^Department of Health Services Research, University of Liverpool, Liverpool L69 3GN, UK; ^2^School of Dentistry, University of Liverpool, Liverpool L3 5PS, UK; ^3^Department of Biostatistics, University of Liverpool, Liverpool L69 3BX, UK; ^4^Department of Women's and Children's Health, University of Liverpool, Liverpool L8 7SS, UK; ^5^Division of Reproductive Health, The University of Warwick, Coventry CV4 7AL, UK; ^6^Centre for Women's Health, The University of Manchester, Manchester M13 9WL, UK

## Abstract

The aim of this study was to investigate the purported link between oral hygiene and preterm birth by using image analysis tools to quantify dental plaque biofilm. Volunteers (*n* = *91*) attending an antenatal clinic were identified as those considered to be “at high risk” of preterm delivery (i.e., a previous history of idiopathic preterm delivery, case group) or those who were not considered to be at risk (control group). The women had images of their anterior teeth captured using quantitative light-induced fluorescence (QLF). These images were analysed to calculate the amount of red fluorescent plaque (Δ*R*%) and percentage of plaque coverage. QLF showed little difference in Δ*R*% between the two groups, 65.00% case versus 68.70% control, whereas there was 19.29% difference with regard to the mean plaque coverage, 25.50% case versus 20.58% control. A logistic regression model showed a significant association between plaque coverage and case/control status (*P* = 0.031), controlling for other potential predictor variables, namely, smoking status, maternal age, and body mass index (BMI).

## 1. Introduction

Early in the 20th century, it was claimed that “there is no relationship between the teeth and the uterus, hence a dental operation cannot have an untoward effect upon pregnancy…” [1]. This position was however already out of date since there had been anecdotal evidence since the late 19th century that gingivitis appeared to be associated with pregnancy [2], an association that was later proven [3]. There are conflicting reports as to whether or not pregnancy induces changes in the host inflammatory response of the periodontium, including levels of tissue type plasminogen activator, interleukin-1*β*, or tumour necrosis factor-*α*, which could in turn be specifically responsible for the observed increase in gingival inflammation [4, 5]. The changes that do occur to the gingiva during pregnancy are at least contributed to by systemic oedema which manifests as an increase in vascular flow along with an increase in the flow rate of gingival crevicular fluid (GCF) [6]. It is likely that the periodontal disease symptoms resulting from plaque accumulation are superimposed on pregnancy-associated physiologic alterations to the gingiva. Importantly, these changes are reversible postpartum.

It was first suggested in 1993 that periodontal disease could influence poor obstetric outcomes such as preterm birth and low birth weight [7]. A number of subsequent studies corroborated this association [8–10], although many other studies found no association [11–14]. One systematic review on the subject concluded that additional longitudinal, epidemiological and interventional studies are required in order to establish whether or not there is a causal relationship between poor dental health and pregnancy outcome [15]. A subsequent systematic review demonstrated risk ratios linking periodontal disease, as defined by a number of defined clinical measures, and preterm birth of between 1.70 (1.03, 2.81) and 1.96 (1.32, 2.90) whilst for preterm birth combined with low birth weight of between 3.06 (1.53, 6.08) and 3.57 (1.87, 6.84) (95% confidence intervals given in parentheses), although the authors state that “this finding should be treated with great caution until the sources of heterogeneity can be explained” [16]. A more recent systematic review found that maternal periodontitis is “modestly but independently associated with adverse pregnancy outcomes,” but such meta analyses are limited by the variability in the different study populations [17]. Another cohort study which utilised oral health questionnaires found that mothers who had had preventative or urgency-based dental treatment within 12 months had a negative association with preterm birth, whilst mothers who considered their own oral health to be “poor” had a positive association with preterm birth [18]. These findings suggested that oral health, rather than periodontal health *per se*, could potentially be linked to preterm birth. It is however unlikely that there is a “one size fits all” relationship between oral health/periodontal intervention and preterm birth due to each individual's particular oral microbiome and immune response [19].

The abundance of maternal oral biofilm has also been studied as a possible presumptive factor for preterm birth. However, as with the periodontal measures, there are studies that report a link [20] and those that discount one [21, 22]. There are a number of different plaque assessment methods available, including those indices which focus on the periodontal tissue [23]. A perceived problem with visual plaque indices is their subjectivity. A number of studies have compared and contrasted their strengths and weaknesses, but it is generally considered that planimetric/photographic methods offer the best reproducibility [24, 25] due to their inherent lack of subjectivity. There is a strong correlation between plaque measurements and gingival indices [26] which suggests that reliable measurements of oral biofilm abundance on the surface of the teeth can be used to infer the periodontal status of the subject.

Quantitative light-induced fluorescence pro (QLF) (Inspektor Research Systems BV, Amsterdam, The Netherlands) is a portable, chair-side device that was developed as a tool to assist clinicians in visualising precarious lesions and quantifying the corresponding changes in enamel mineral density [27]. The technique works by assessing the intrinsic fluorescence of vital tooth enamel during illumination with violet light. The tooth fluorescence is collected via a modified dental mirror which incorporates a video camera and a proprietary filter, whilst the camera is in turn connected to a computer for data collection, image repositioning assistance, and subsequent analysis using appropriate software. Although QLF can detect mature oral biofilm growing near the gingival margin due to the red fluorescence emitted by bacterial porphyrins [28], this system is not specifically optimised for this purpose. QLF can however be used to provide a rapid assessment of plaque density when employed in conjunction with disclosing agents [29]. When used in this modality, QLF is essentially used as an intraoral camera, whilst the proprietary software captures and records the images whilst proving the user assistance in repositioning the camera between subsequent imaging sessions. QLF is also thought to be particularly effective in prompting behavioural change in oral hygiene practices [30]. QLF could be a powerful assessment tool in the area of public health, specifically of dental health in pregnancy, but has not yet been utilised in this arena.

The aim of this project was to evaluate the use of QLF to measure dental plaque biofilm coverage of the teeth in a case control study of pregnant women on the assumption that increasing levels of plaque correspond with declining gingival health which in turn is purported to be linked to birth outcome. This would build upon previous studies which evaluated plaque abundance [20–22] in relation to preterm birth. The volunteers in the present study will not be asked to undergo a periodontal examination in order to maintain the nonintervention status of the project and to maximise participation rates. The null hypothesis was that heavy plaque biofilm coverage was not associated with an increased risk of preterm birth.

## 2. Materials and Methods

### 2.1. Recruitment

The recruitment criteria for this study were women in the 15–20th week of their pregnancy attending for antenatal care at the Liverpool Women's National Health Service Foundation Trust. The recruitment process and subsequent investigations of this observational study were ratified by the Liverpool National Health Service Research Ethics Committee (05/Q1505/148). It is standard clinical practice for the consultant obstetrician to identify those women who they consider to be at risk of preterm birth, specifically those with a history of previous premature delivery, preeclampsia, or had recently given birth. This triage allows for more focussed and more frequent clinical observation of patients during their pregnancy. Following a discussion with maternity staff, the volunteers were provided with an information sheet, oral health questionnaire, and a consent form. The women who volunteered for this study were assigned to one of the two groups; those who were considered to be “at risk” of preterm delivery (case group) and those who were not considered to be at risk (control group). The allocation of individuals to either group for the purposes of the present study mirrored their existing clinical consideration. The volunteers attended their antenatal appointments in the afternoon as normal where routine medical information was recorded, including their body mass index (BMI), age, and smoking status, before they attended their oral examination in an adjacent clinic. The researchers who captured and analysed the QLF images were blinded as to the case/control status of the volunteers. The data was pseudoanonymised throughout the study by identifying the volunteers by their hospital patient number.

### 2.2. QLF Image Capture

The examinations were conducted in a darkened room in order to maximise the quality of the QLF images captured [31]. The volunteer was first offered the use of a cotton roll containing Yellow Soft Paraffin BP (Ecolab LTD., Garforth, Leeds) to protect their lips during the imaging session. A sterile retractor was fitted to expose the volunteer's anterior teeth before QLF images were captured of the 12 buccal surfaces of the incisors and canines (FDI 13–23 and 33–43) (QLF Pro, Inspektor Research Systems BV, Amsterdam, The Netherlands). Following image capture, the 12 teeth were painted (Microbrush, regular size, Grafton, USA/Clogherane, Dungarvan, Co. Waterford, Ireland) with a disclosing agent (PlaqSearch, OralDent, Kimbolton, UK). The volunteer was asked to gently rinse their mouth (Tellodont, Gargle and Mouth Wash Tablets, Tell Products LTD., London, UK) and expectorate into a sink before a second set of QLF images were recorded. The QLF Pro's image repositioning system was used to achieve the same angulation between the undisclosed and disclosed images. The volunteer was offered a new toothbrush and a portion of toothpaste and invited to clean their teeth before leaving the clinic.

### 2.3. Image Analysis

The red fluorescence of plaque in the undisclosed QLF images was analysed using the proprietary software associated with the unit (Inspektor-Pro 2.0.0.34). Briefly, an elliptical area on the tooth without biofilm was selected as a reference before a user-defined region of interest (ROI) was drawn around the boundary of the tooth. A software algorithm then returned the area (in mm^2^) of red fluorescence within the ROI along with a value for the percentage difference in red fluorescence (Δ*R*%) when compared with the plaque free-reference [32].

**Figure 1:**
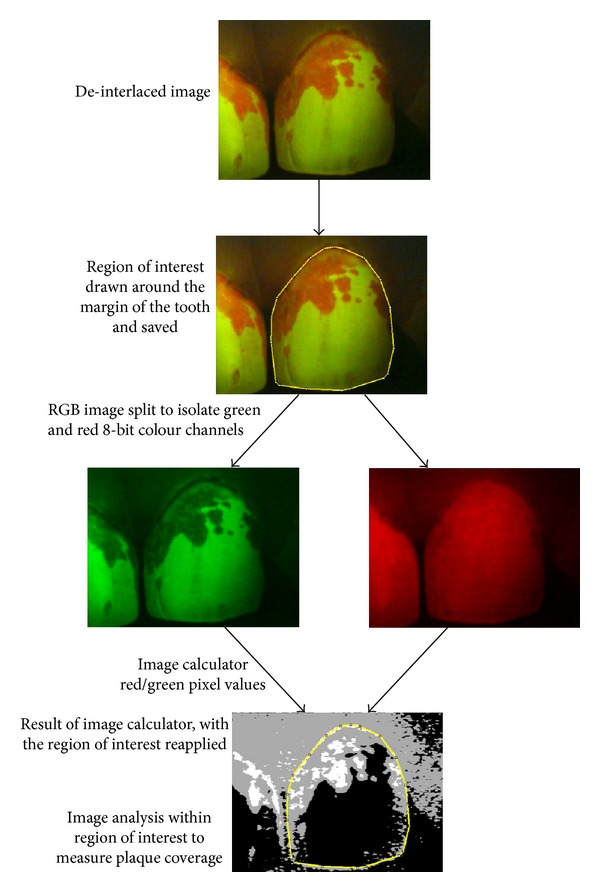
The process of measuring the percentage disclosed plaque coverage of teeth using ImageJ.

**Figure 2:**
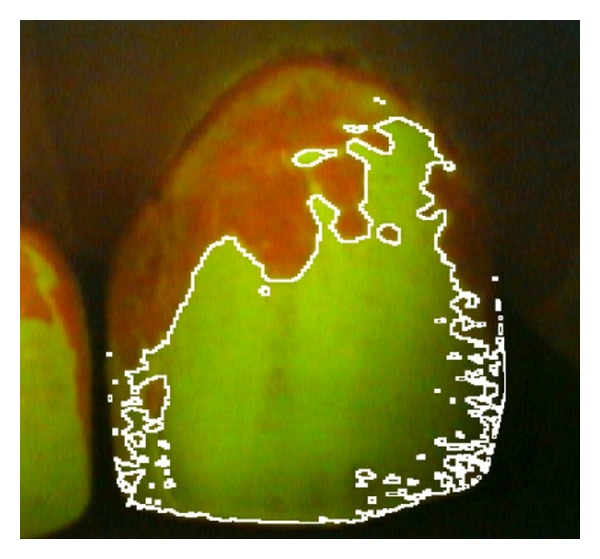
Superimposition of the original QLF image and the boundary between the areas with and without plaque as determined by ImageJ (shown in white) to demonstrate the degree of conformity.

Plaque coverage was determined using the images of disclosed plaque which were analysed with the open source, Java-based image analysis program ImageJ (v1.47c, National Institutes of Health, USA; http://rsb.info.nih.gov/ij/). Prior to analysis, the images were processed by the “deinterlace” filter (http://rsbweb.nih.gov/ij/plugins/de-interlace.html) to remove any horizontal interlacing artefacts which may have emanated from the QLF Pro video capture system. The “polygon selection” tool was then used to apply a ROI which encompassed the tooth being analysed, which was then saved as a “selection.” Next, the image was split into its component colour channels (RGB; red/green/blue) to yield three monochromatic, 8-bit images following which the “image calculator” function was used to divide the pixel brightness values from the red image by that of the green image. The result of this calculation yielded an almost entirely black image output, which nevertheless contained pixel brightness values of 0, 1, 2, and occasionally 3 from the 8-bit range (0–255; 0 = black, 255 = white). At this stage the differences between these “dark” pixels could be visualised by adjusting the maximum value of the “brightness/contrast” function; however this process was not necessary for image analysis and it did not alter the raw numerical data (Figure 1). The ROI which had previously been saved was imported back into the result of the image calculator and the “histogram” function was then used to count the number of pixels of each brightness value within it. The total number of pixels within the ROI was used to calculate the percentage plaque coverage by allocating pixels with a value of “0” as “plaque-free” and those with a value ≥1 as “plaque covered.” The *ad hoc* superimposition of original images with the binary results of the image calculator showed good conformity (Figure 2).

## 3. Statistical Analysis

The mean percentage of plaque coverage and red fluorescence was calculated for the anterior teeth of each volunteer before their status as “case” or “control” was revealed. Women who had less than 10 natural anterior teeth (i.e., not crowns, bridges, or veneers) and those who had recently taken antibiotics were excluded from the final analyses.

The association of the measurements with the case/control status of the participants was assessed using multiple logistic regression analysis. The model controlled for the well-established confounding effects of smoking during pregnancy [33] and body mass index (BMI) of the mother [34] upon preterm birth.

The relationship between plaque coverage (% area) and red fluorescence (Δ*R*%) was calculated by the Pearson product-moment correlation coefficient.

## 4. Results

A total of 109 volunteers were recruited into this study; 7 were excluded due to having less than 10 out of 12 natural anterior teeth present whilst a further 9 were excluded during the data analysis stage on account of antibiotic usage or incomplete data with regard to their BMI or smoking status. In addition, two volunteers withdrew themselves from the study during the imaging process due to nausea. The data presented henceforth refers to the remaining 91 volunteers comprising 51 “cases” and 40 “controls.”

**Figure 3:**
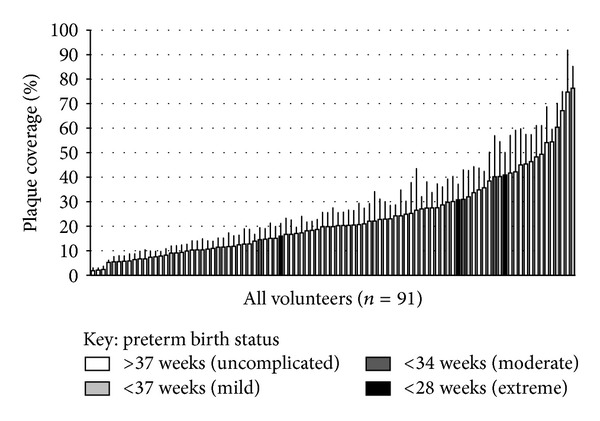
Ranked distribution of mean percentage plaque coverage for all volunteers highlighting those who gave birth preterm. Error bars show 95% confidence intervals for the measured teeth (*n* = 10–12).

The characteristics of the study participants are summarised in Table 1. *T*-tests or chi-squared test, where relevant, did not reveal a statistically significant difference between the case and control groups for plaque coverage, red fluorescence, or any of the potentially confounding measures. Image analysis revealed a 19.29% difference with regard to the mean plaque coverage, 25.50% case versus 20.58% control. A logistic regression model revealed a significant association between plaque coverage and case/control status (*P* = 0.031) (Table 2), controlling for other potential predictor variables (red fluorescence, smoking status, maternal age, and BMI). As a purely descriptive statistic, taking the distribution of the entire volunteers and identifying those individuals whose birth outcome was preterm, defined as less than 37 weeks, suggest that they had a tendency to have heavier plaque coverage (Figure 3).

There was only a 5.39% difference in Δ*R*% between the two groups, 65.00% case versus 68.70% control, which the logistic regression model revealed as significant (*P* = 0.042).

**Figure 4:**
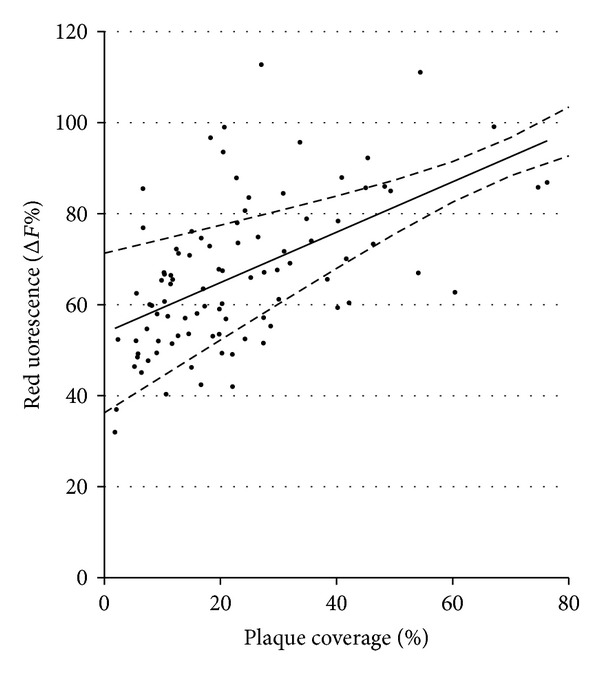
Pearson product-moment correlation coefficient (0.55) between plaque coverage (%) and red fluorescence (Δ*R*%) showing line of best fit (black line) and 95% confidence intervals (dashed lines).

The Pearson product-moment correlation coefficient between plaque coverage (% area) and red fluorescence (Δ*R*%) gave an *r* value of 0.55, which is generally considered to be indicative of an association (Figure 4). This result indicates that red fluorescence increases as plaque coverage increases, although a directly proportional relationship probably does not adequately describe the actual situation.

## 5. Discussion

**Figure 5:**
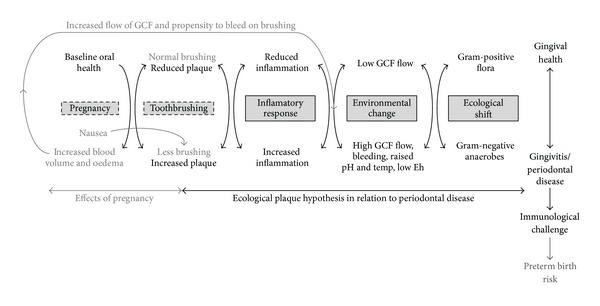
Proposed interrelationship between the changes occurring in the maternal gingiva superimposed upon changing tooth brushing habits during pregnancy and their interaction with the plaque environment and microbiota as proposed in the ecological plaque hypothesis. Eh, redox potential. Figure adapted with permission from [35].

Dental plaque is a dynamic microbial community whose composition changes in response to the local environment. The causality and consequences of these shifts in community composition are described in the ecological plaque hypothesis [35] which offers a model for how microbial homeostasis can be disrupted in such a way as to lead to caries or periodontal disease. This hypothesis suggests that changes known to take place in the gingivae of pregnant women, an increased flow of gingival crevicular fluid [6] and propensity to bleed on brushing due to increased blood volume [22], will influence plaque homeostasis. This influence could also be exacerbated by nausea during pregnancy [36], which according to comments from a number of volunteers caused them to brush less than they would have normally done. This reduction of brushing, increased GCF, and gingival bleeding will superimpose upon the ecological plaque hypothesis model and shift the oral microbiota towards the Gram-negative anaerobic community that is associated with periodontal disease (Figure 5), which may be associated with the risk of preterm birth. A number of putative periodontal pathogens, *Prevotella *spp. and *Porphyromonas gingivalis*, have been isolated in cases of bacterial vaginosis and a direct association has been observed between vaginosis and gingivitis [37].

However, an important point needs to be taken into account when considering using plaque coverage as a measure of oral health, specifically periodontal health. The relationship between the amount of plaque covering the teeth and host inflammatory response cannot be considered to be linear [38]. Furthermore, host genetics play a vital role in determining the development and sequelae of periodontal disease [39], which means that in a population who employs no oral hygiene measures, some may develop periodontitis, whilst the others do not [40]. Notwithstanding this, it has been shown that the amount of plaque influences the proportions of individual bacterial species within it [41], including members of the “orange complex” (i.e., species that are associated with periodontitis) [42].

The present study was carried out on the assumption that increasing plaque coverage of the teeth and increasing red fluorescence of plaque were associated with declining oral hygiene. Therefore, if poor oral health is indeed linked to preterm birth, then it would be expected that increased plaque coverage and red fluorescence would be present in those mothers who delivered early. This association was alluded to in the means of the plaque coverage data and confirmed in this observational study using a logistic regression model which tested plaque coverage against case/control status which controlled for smoking status, age, and BMI of the mother. Parity (whether or not the volunteer has given birth previously) could not be included into the model because the primary indicator for allocating a woman into either the case or control group was a previous idiopathic preterm birth. The logistic regression model could not be applied upon the actual birth outcome data, as opposed to their perceived “at risk” status, since the number of women who actually delivered preterm was insufficient to perform any meaningful statistical analysis. Of the 91 volunteers, only 17 (18.68%) delivered prematurely (<37 weeks). When only problematic preterm births (<34 weeks) were considered, this sample size fell to only 6 (6.59%). The birth outcome data from one individual in the control group was not available. Extrapolating the participation rates found in this study, which was conducted at a weekly antenatal clinic, it would take several years to gather a data set of sufficient size to allow meaningful statistical analyses to be undertaken using data for this thankfully rare birth outcome. If this research is pursued in future it would best be conducted as a multicentre collaboration involving antenatal clinics where high risk cases are treated. The descriptive analysis of the case control data suggests that such a study would be worthwhile (Figure 3).

Due to the subjective nature of manually selecting ROIs around the teeth during image analysis, the percentage of plaque coverage of a particular tooth (shown in Figure 2) was measured independently by two individuals (Qian Wang and Christopher K. Hope) to give a representative measure of reproducibility. The two values for percentage plaque coverage in this instance were measured as 43.01% and 42.36% (1.52% difference). This suggests that the subjectivity of individual researchers is not an issue for these analyses.

The relatively small proportion of patients attending the antenatal clinic who then consented to volunteer for this project was a considerable problem for this study, especially with regard to the “control” group. This disparity was assumed to be due to the “case” group including a number of women who had had a previous idiopathic premature delivery and as such had a personal motivation to participate, whereas “control” volunteers did so altruistically [43]. It is also likely that many women simply chose not to participate in this study because doing so would prolong the time they had already spent in the antenatal clinic. This effect may have been exacerbated because the prospective volunteers were asked to participate in a “dental study” to “photograph their teeth”; a number of women would undoubtedly have declined either due to dental anxiety or embarrassment at their dentition or oral hygiene in their pregnant state which may have declined for the reasons already alluded to. Participation rates would probably have been even lower if the study had also involved a dental examination. These perceived problems were apparent in the fact that only ~20% of the volunteers who returned to the antenatal clinic for subsequent follow-up appointments with obstetricians consented to additional QLF image capture sessions. However, to avoid any potential bias due to the Hawthorne effect [44], which in this situation could manifest as a subconscious improvement in the oral hygiene measures in advance of an antenatal/plaque imaging appointment, women who had multiple measurements taken only had their first set of images included in the analysis to match the majority of volunteers. Participation may be improved in future studies by offering a dental inspection as part of the screening procedure as well as offering advice on how to control plaque during pregnancy. It would also be prudent to remind the volunteers of the importance of their oral health and that dental treatment was free during their pregnancy as well as twelve months postpartum.

The QLF Pro system used in this study was optimised to visualise differences in enamel fluorescence as a result of areas of demineralisation. This has recently been superseded by “QLF digital” (QLFD) which utilises a digital SLR camera incorporating both white and violet (405 nm) light-emitting diodes together with an upgraded filter system. QLFD is designed to optimise the visualisation and quantification of dental plaque by increasing the contrast between the green fluorescing tooth and red fluorescing plaque. A future study would benefit from utilising QLFD technology to make it more acceptable to pregnant women attending antenatal clinic since it is much quicker than QLF pro (which images individual teeth and surfaces using a hand-piece style intraoral camera) and far less invasive since it is analogous to taking a photograph. Although QLFD cannot easily be used to image molars in its current form, it would be ideal for obtaining a more reliable measure of plaque fluorescence on the anterior teeth, as examined in this study. The process could also be made more acceptable to volunteers by asking them to chew a disclosing tablet, rather than allowing a researcher to paint the agent onto the teeth. Also, improvements and developments in plaque imaging via bacterial fluorescence may obviate the need for disclosing agents at all since fluorescence alone may give a reliable measurement of total plaque coverage. If patients are shown images of their own plaque covered teeth as part of this process it may give them added impetus to maintain good oral hygiene throughout their pregnancy, despite feeling nauseous and frequently experiencing bleeding gingivae on brushing.

The observed negative correlation between red fluorescence and preterm birth is difficult to explain since increased Δ*R*% values were associated with the control group as opposed to the case group, the opposite of that found with the percentage of plaque coverage data. A possible explanation is that although the red fluorescence observed with QLF is associated with the accumulation of significant levels of plaque and an abundance of porphyrin-producing Gram-negative anaerobes near the gingival margin, there is no credible mechanism as to why such fluorescence would continue to increase as additional plaque forms distal to the gingiva in individuals with high plaque coverage values. So although plaque coverage is an indicator of oral hygiene status that could potentially be used as an indicator of preterm birth risk, plaque fluorescence does not appear to be a useful predictor at this time.

With the correct equipment, it is relatively simple to determine the area of a tooth that is covered with dental plaque, although such analyses cannot reveal the bacterial composition of the biofilm, microbial density or measure the corresponding host response in terms of gingival inflammation. A much larger sample size would be required to incorporate the birth outcomes into the multiple regression analysis. The inclusion of other disease indicators, such as gingivitis, probing depth, or attachment loss, would be a useful addition to the study, although this would undoubtedly affect patient recruitment and such studies are not novel without the inclusion of planimetric plaque assessment methods, such as QLF. Recent technological developments such as QLFD would help facilitate the collection of a larger data set with less inconvenience to the volunteers in the future.

## 6. Conclusions

QLF and image analysis tools can be used to measure plaque coverage of the teeth. A linear regression model indicated that there was an association between plaque coverage and those women who were considered to be at risk of preterm birth. This association accounted for confounding factors associated with preterm birth: smoking, age, and BMI. Pregnant women should be advised of the importance of maintaining good oral hygiene throughout their pregnancy.

## Figures and Tables

**Table 1:** Summary of red fluorescence and plaque coverage data of the anterior teeth (*n* = 10–12 per individual) with other logistic regression model parameters (figures in parentheses are standard deviations unless identified as percentages).

Variable	Case	Control	*P* values comparing case and control
Sample size (*n*)	51	40	
Mean plaque coverage (%)	25.50 (17.45)	20.58 (14.39)	0.154^†^
Mean red fluorescence (Δ*R*%)	65.00 (16.42)	68.70 (16.61)	0.292^†^
Mean maternal age (years)	28.69 (5.91)	30.78 (6.41)	0.110^†^
≤35 (*n*)	42 (82%)	33 (83%)	0.985^‡^
>35 (*n*)	9 (18%)	7 (18%)	
Smoking status			
Nonsmoker (*n*)	31 (61%)	30 (75%)	0.105^‡^
Stopped during pregnancy (*n*)	5 (10%)	4 (10%)	
Smoker during pregnancy (*n*)	15 (29%)	6 (15%)	
BMI before pregnancy	26.05 (5.29)	26.21 (5.32)	0.882^†^
Underweight (BMI < 19) (*n*)	2 (4%)	1 (3%)	0.910^‡^
Normal (BMI 19–25) (*n*)	24 (47%)	20 (50%)	
Overweight (BMI > 25) (*n*)	25 (49%)	19 (48%)	
Delivery outcome*			
<28 weeks (extreme) (*n*)	2 (4%)	0	
≥28–<34 weeks (moderate) (*n*)	2 (4%)	1 (3%)	
≥34–<37 weeks (mild) (*n*)	10 (20%)	1 (3%)	
≥37 weeks (*n*)	37 (73%)	37 (95%)	

^†^ *t*-test; ^‡^chi-squared test; *one delivery outcome missing from the control group.

**Table 2:** Results of multiple regression analysis controlling for the confounding effects of smoking, maternal age, and BMI.

Variable	*β*	Std Error	Sig.	OR	95% CI for OR	
					Lower	Upper
Plaque coverage (%)	−0.042	0.019	0.031*	0.959	0.923	0.996
Red fluorescence (Δ*R*%)	0.036	0.018	0.042*	1.037	1.001	1.073
Smoking	−0.822	0.579	0.156	0.439	0.141	1.368
BMI	0.005	0.045	0.913	1.005	0.920	1.098
Maternal age	0.030	0.039	0.439	1.030	0.955	1.111

Key: *β*: standardised coefficients; OR: odds ratio; CI: 95% confidence intervals; *indicates statistical significance (*P* < 0.05).
